# Supplementary material for: Amplifiers of selection for the Moran process with both Birth-death and death-Birth updating
Source: PLoS Comput Biol. 2024 Mar 29;20(3):e1012008. doi: 10.1371/journal.pcbi.1012008 (PMC11006194; doi:10.1371/journal.pcbi.1012008)
Supplement: S1 Appendix — (PDF) [file pcbi.1012008.s001.pdf]

Supplementary Information: Amplifiers for both Birth-death and death-Birth updating

Jakub Svoboda[1], Soham Joshi[1], Josef Tkadlec[2], and Krishnendu Chatterjee[1]

[1]IST Austria, Klosterneuburg, Austria
[2]Computer Science Institute, Charles University, Prague, Czech Republic

Contents

1 Preliminaries **1**

2 Negative result 2 **2**

3 Negative result 3 **3**

4 Positive result **5**
- 4.1 Auxiliary statements 5
- 4.2 Construction 5
- 4.3 Proof of Amplification 7

This is a supplementary information to the manuscript *Amplifiers for both Birth-death and death-Birth updating*. It contains formal proofs of the theorems listed in the main text.

1 Preliminaries

Given an undirected graph $G_N = (V, E)$ on N nodes, the *degree* of a node u, denoted $\deg(u)$, is the number of neighbors of u in G_N. When the edges are weighted, we define the degree $\deg(u) = \sum_{v:(u,v)\in E} w(u, v)$ as the sum of the weights of all the adjacent edges. As a direct extension of [3, 4, 10] and as noted in [1] we obtain the following formula for fixation probability under neutral drift ($r = 1$). For completeness, we include a proof.

Lemma 1 (Fixation probability on edge-weighted undirected graphs when $r = 1$)**.** *Let $G_N = (V, E)$ be an edge-weighted undirected graph on N nodes and $S \subset V$ any set of vertices occupied by mutants. Then*

$$\rho^{\mathrm{Bd}}_{r=1}(G_N, S) = \frac{\sum_{u\in S} 1/\deg(u)}{\sum_{v\in V} 1/\deg(v)} \qquad \text{and} \qquad \rho^{\mathrm{dB}}_{r=1}(G_N, S) = \frac{\sum_{u\in S} \deg(u)}{\sum_{v\in V} \deg(v)}.$$

Proof. Let $p_{u\to v}$ be the probability that, in a single step, an individual at node u produces an offspring that replaces an individual at node v. For Birth-death updating, it suffices to check that for any subset $S \subset V$ of mutant nodes and any edge (u, v) connecting a mutant node $u \in S$ and a non-mutant node $v \notin S$ we have

$$p_{u\to v} \cdot \frac{1/\deg(v)}{\sum_{v'\in V} 1/\deg(v')} = p_{v\to u} \cdot \frac{1/\deg(u)}{\sum_{v'\in V} 1/\deg(v')}.$$

Since for Birth-death updating and $r = 1$ we have $p_{u\to v} = \frac{1}{N} \cdot \frac{w(u,v)}{\deg(u)}$, both sides rewrite as

$$\frac{\frac{1}{N} \cdot \frac{w(u,v)}{\deg(u)\deg(v)}}{\sum_{v'\in V} 1/\deg(v')},$$

and so the claim is proved. Likewise, for death-Birth updating it suffices to check that

$$p_{u\to v}\cdot\frac{\deg(v)}{\sum_{v'\in V}\deg(v')}=p_{v\to u}\cdot\frac{\deg(u)}{\sum_{v'\in V}\deg(v')}.$$

Since for death-Birth updating and $r=1$ we have $p_{u\to v}=\frac{1}{N}\cdot\frac{w(u,v)}{\deg(v)}$, this time both sides rewrite as

$$p_{u\to v}\cdot\frac{\frac{w(u,v)}{N}}{\sum_{v'\in V}\deg(v')}.$$

□

The proof of our positive result relies on three existing results. For convenience, we list them here. First, there exist unweighted graphs called *Incubators* that are strong amplifiers under Birth-death updating [8, Theorem 2].

Lemma 2. *There exists a family of graphs $\mathcal{A}_N^{Bd}$ such that for all $r>1$, we have*

$$\rho_r^{\mathrm{Bd}}(\mathcal{A}_N^{Bd})\geq 1-\mathcal{O}(N^{-1/3}).$$

Second, there exist edge-weighted graphs called *Separated Hubs* that are substantial amplifiers under death-Birth updating [2, Theorem 3].

Lemma 3. *There exists a family of graphs $\mathcal{A}_N^{dB}$ such that for all $r>1$, we have*

$$\rho_r^{\mathrm{dB}}(G_N)=\frac{N}{2N+1}\cdot\frac{1-\frac{1}{r^3}}{1-\frac{1}{r^{3N}}}.$$

Third, the evolutionary dynamics terminates polynomially quickly in terms of the population size N, under both the Birth-death updating [5, Theorem 9] and the death-Birth updating [7, Theorem 1].

Lemma 4. *Fix $r>1$. For Bd and dB process on an undirected graph with N vertices with the highest ratio between edge weights $\frac{1}{\varepsilon}$, the expected fixation time is in $\mathcal{O}(\frac{N^4}{\varepsilon})$.*

2 Negative result 2

In this section, we show that one fixed neutral mutant cannot have a better fixation probability in both processes than on a complete graph. This means that even if we can choose the starting position, we are not guaranteed to increase the fixation probability for both processes.

Theorem 1. *Let G_N be a graph and v an initial mutant node. Then at least one of the following is true:*

1. $\rho_{r=1}^{\mathrm{Bd}}(G_N,v)<\rho_{r=1}^{\mathrm{Bd}}(K_N)$;
2. $\rho_{r=1}^{\mathrm{dB}}(G_N,v)<\rho_{r=1}^{\mathrm{dB}}(K_N)$;
3. $\rho_{r=1}^{\mathrm{Bd}}(G_N,v)=\rho_{r=1}^{\mathrm{Bd}}(K_N)$ *and* $\rho_{r=1}^{\mathrm{dB}}(G_N,v)=\rho_{r=1}^{\mathrm{dB}}(K_N)$.

Proof. First, note that $\rho_{r=1}^{\mathrm{Bd}}(K_N)=\rho_{r=1}^{\mathrm{dB}}(K_N)=1/N$. Next, recall the known formulas for the fixation probability on undirected graphs under neutral drift (see Lemma 1 and [4, 10]], namely:

$$\rho_{r=1}^{\mathrm{Bd}}(G_N,v)=\frac{1/\deg(v)}{\sum_{u\in V}1/\deg(u)}\quad\text{and}\quad\rho_{r=1}^{\mathrm{dB}}(G_N,v)=\frac{\deg(v)}{\sum_{u\in V}\deg(u)}.$$

As the final ingredient, note that for any N non-negative numbers $x_1,\ldots,x_N$ we have a bound

$$\left(\frac{1}{x_1}+\frac{1}{x_2}+\cdots+\frac{1}{x_N}\right)\cdot(x_1+x_2+\cdots+x_N)\geq N^2.$$

This follows e.g. from the inequality between the arithmetic and harmonic mean of numbers $x_1, \dots, x_N$ (called AM-HM), or from Cauchy-Schwarz inequality. Moreover, the equality occurs if and only if $x_1 = x_2 = \cdots = x_N$. Applying this bound to $x_i = \deg(v_i)$ we obtain

$$\rho^{\mathrm{Bd}}_{r=1}(G_N, v) \cdot \rho^{\mathrm{dB}}_{r=1}(G_N, v) = \frac{1/\deg(v)}{\sum_{u\in V} 1/\deg(u)} \cdot \frac{\deg(v)}{\sum_{u\in V} \deg(u)} = \frac{1}{\left(\sum_{u\in V} 1/\deg(u)\right)\cdot\left(\sum_{u\in V}\deg(u)\right)} \le \frac{1}{N^2}.$$

If equalities occur everywhere then $\deg(v_1) = \cdots = \deg(v_N)$, thus $\rho^{\mathrm{Bd}}_{r=1}(G_N, v) = \rho^{\mathrm{dB}}_{r=1}(G_N, v) = 1/N$. Otherwise, the product is strictly less than $1/N^2$, thus at least one of $\rho^{\mathrm{Bd}}_{r=1}(G_N, v)$ and $\rho^{\mathrm{dB}}_{r=1}(G_N, v)$ is strictly less than $1/N$. □

The following example illustrates that there exists a graph and a subset $S = \{u, v\}$ of $k = 2$ nodes, such that the fixation probability starting from mutants at both u and v is strictly greater than fixation probability starting from $k = 2$ mutant nodes on a well-mixed population, both for the Birth-death and for the death-Birth updating.

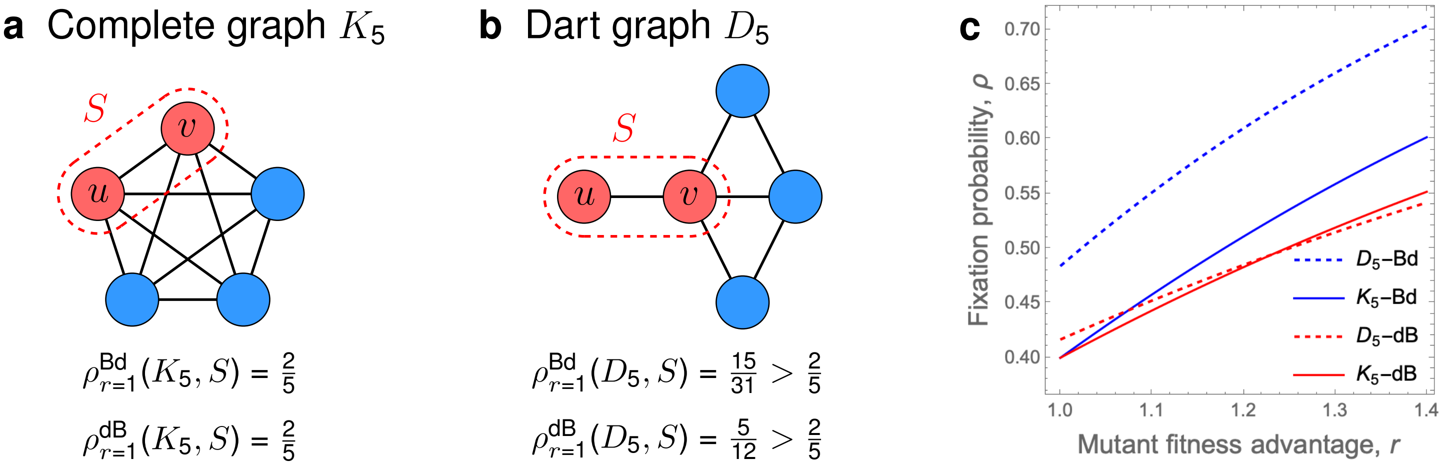

Figure 1: **Mutant subset that amplifies for both Bd and dB. a,** With two neutral mutants ($r = 1$) on a complete graph K_N, the fixation probability is equal to $2/N$ under both Birth-death and death-Birth updating. **b,** When two neutral mutants initially occupy vertices u and v of the so-called dart graph D_5, the fixation probability under both Birth-death and death-Birth updating is increased. **c,** As r increases above roughly $r \approx 1.24$, the fixation probability on the Dart graph under death-Birth updating drops below the reference value of two mutants on a complete graph K_5. Under Birth-death updating, the effect persists for $r \ge 1$. (Data obtained by numerically solving the underlying Markov chains.)

The intuition behind the result is that node u is a really good initial mutant node for Birth-death updating, and node v is a really good initial mutant node for death-Birth updating. Together, they form an above-average set of two mutant nodes, even when compared to a complete graph with two initial mutants.

3 Negative result 3

In this section, we prove that for any fixed vertex, in the first step, the ratio between increasing and decreasing the number of mutants cannot be better than in the complete graph in both processes. This means we cannot find a vertex from which both processes spread better than in the complete graph. To achieve amplification for both processes, we know that some vertices will be better for Bd and some for dB amplification.

Recall that given Moran Birth-death process run on graph G_N with an initial mutant node u, the quantity $\gamma^{\mathrm{Bd}}_r(G_N, u)$ is the probability that the first reproduction event that changes the size of the mutant subpopulation is the initial mutant reproducing (as opposed to the initial mutant being replaced by one of its neighbors). Similarly, we define $\gamma^{\mathrm{dB}}_r(G_N, u)$ for the death-Birth process.

Theorem 2. *Let G_N be a graph, u an initial mutant node, and $r \ge 1$. Then at least one of the following is true:*

1. $\gamma^{\mathrm{Bd}}_r(G_N, u) < \gamma^{\mathrm{Bd}}_r(K_N)$;

2. $\gamma_r^{\mathrm{dB}}(G_N,u) < \gamma_r^{\mathrm{dB}}(K_N)$*;*

3. $\gamma_r^{\mathrm{Bd}}(G_N,u) = \gamma_r^{\mathrm{Bd}}(K_N)$ *and* $\gamma_r^{\mathrm{dB}}(G_N,u) = \gamma_r^{\mathrm{dB}}(K_N)$*.*

Proof. Denote by $T(u) = \sum_{v\colon (u,v)\in E} \frac{w(u,v)}{\deg v}$ the so-called *temperature* of node u, that is, the rate at which node u is replaced by its neighbors in the neutral case.

Denote by $p^+_{\mathrm{Bd},r} = p^+_{\mathrm{Bd},r}(G_N,u)$ the probability that in a single step of the Moran Birth-death process the mutant reproduces, and by $p^-_{\mathrm{Bd},r} = p^-_{\mathrm{Bd},r}(G_N,u)$ the probability that it gets replaced by a resident. Denoting the total fitness by $F = N + (r-1)$ we have

$$p^+_{\mathrm{Bd},r} = \frac{r}{F} \quad \text{and} \quad p^-_{\mathrm{Bd},r} = \sum_{v\colon (u,v)\in E} \frac{1}{F}\cdot\frac{w(u,v)}{\deg v},$$

and thus

$$\gamma_r^{\mathrm{Bd}}(G_N,u) = \frac{p^+_{\mathrm{Bd},r}}{p^+_{\mathrm{Bd},r} + p^-_{\mathrm{Bd},r}} = \frac{r}{r + \sum_{v\colon (u,v)\in E}\frac{w(u,v)}{\deg v}} = \frac{r}{r+T(u)}.$$

In particular, in the complete graph K_N each node has temperature 1, and thus

$$\gamma_r^{\mathrm{Bd}}(K_N) = \frac{r}{r+1}.$$

If $T(u)\ge 1$ then $r/(r+T(u)) \le r/(r+1)$ and hence $\gamma_r^{\mathrm{Bd}}(G_N,u) \le \gamma_r^{\mathrm{Bd}}(K_N)$ with equality if and only if $T(u)=1$. From now on, suppose $T(u)\le 1$.

Consider Moran death-Birth process and define the quantities $p^+_{\mathrm{dB},r} = p^+_{\mathrm{dB},r}(G_N,u)$ and $p^-_{\mathrm{dB},r} = p^-_{\mathrm{dB},r}(G_N,u)$ as above. Then

$$p^+_{\mathrm{dB},r} = \sum_{v\colon (u,v)\in E} \frac{1}{N}\cdot\frac{r\cdot w(u,v)}{(r-1)w(u,v)+\deg(v)} \quad \text{and} \quad p^-_{\mathrm{dB},r} = \frac{1}{N},$$

therefore

$$\gamma_r^{\mathrm{dB}}(G_N,u) = \frac{\sum_{v\colon (u,v)\in E}\frac{r\cdot w(u,v)}{(r-1)w(u,v)+\deg(v)}}{1+\sum_{v\colon (u,v)\in E}\frac{r\cdot w(u,v)}{(r-1)w(u,v)+\deg(v)}}.$$

In particular, for the complete graph K_N and any its node u we have

$$\sum_{v\colon (u,v)\in E}\frac{r\cdot w(u,v)}{(r-1)w(u,v)+\deg(v)} = (N-1)\cdot\frac{r}{(r-1)+(N-1)}.$$

Hence in order to prove $\gamma_r^{\mathrm{dB}}(G_N,u)\le\gamma_r^{\mathrm{dB}}(K_N)$, it suffices to prove

$$\sum_{v\colon (u,v)\in E}\frac{r\cdot w(u,v)}{(r-1)w(u,v)+\deg(v)} \le \frac{(N-1)r}{(r-1)+(N-1)}.$$

We rearrange

$$\sum_{v\colon (u,v)\in E}\frac{\frac{w(u,v)}{\deg(v)}}{(r-1)\frac{w(u,v)}{\deg(v)}+1} \le \frac{1}{\frac{r-1}{N-1}+1}.$$

When $r=1$, the desired claim reduces precisely to $T(u)\le 1$. Suppose $r>1$, that is $r-1>0$, and consider a function $f\colon (0,\infty)\to(0,\infty)$ defined by $f(x)=\frac{x}{(r-1)x+1}$. Then f is concave and increasing, therefore by Jensen's inequality we have

$$\sum_{v\colon (u,v)\in E}\frac{\frac{w(u,v)}{\deg(v)}}{(r-1)\frac{w(u,v)}{\deg(v)}+1} \le |N(u)|\cdot\frac{\frac{1}{|N(u)|}\sum_{v\colon (u,v)\in E}\frac{w(u,v)}{\deg(v)}}{(r-1)\frac{1}{|N(u)|}\sum_{v\colon (u,v)\in E}\frac{w(u,v)}{\deg(v)}+1} = \frac{T(u)}{\frac{r-1}{|N(u)|}\cdot T(u)+1},$$

where $|N(u)| = |\{v\colon (u,v)\in E\}|$ is the number of neighbors of u in G.

Finally, since the function f is increasing, using bounds $T(u) \leq 1$ and $|N(u)| \leq N-1$, the right-hand side is at most

$$\frac{T(u)}{\frac{r-1}{|N(u)|} \cdot T(u) + 1} \leq \frac{1}{\frac{r-1}{|N(u)|} + 1} \leq \frac{1}{\frac{r-1}{N-1} + 1}$$

as desired. For the equality to occur in the first step, we must in particular have $T(u) = 1$, in which case the other equality $\gamma_r^{\mathrm{Bd}}(G_N, u) = \gamma_r^{\mathrm{Bd}}(K_N)$ holds too. □

4 Positive result

In this section, we prove the main positive result which states that there exists an undirected, edge-weighted graph that is simultaneously an amplifier of selection for Birth-death Moran process and for death-Birth Moran process (under uniform mutant initialization). We first bound the number of steps until fixation or extinction for both processes (Bd and dB) and any graph. Second, we show that for any graph, there is a good starting vertex where a mutant has fixation probability at least $\frac{1}{N}$. Then we construct the graph and we prove that it is indeed an amplifier for both processes.

4.1 Auxiliary statements

Lemma 5. *For Bd and dB process for any r on an undirected graph with N vertices with the ratio between edge weights at most $\frac{1}{\varepsilon}$, the probability that the process is not completed after $\mathcal{O}(N^5/\varepsilon)$ steps is in $\mathcal{O}(\frac{1}{2^N})$.*

Proof. From Lemma 4, we can take constant c such that for both processes and all graphs with N vertices, the expected time is at most cN^4/ε. From Markov's inequality [9], the probability that the process takes more than $2cN^4/\varepsilon$ steps is at most $\frac{1}{2}$. If the process does not finish, the expected time is again cN^4/ε. That means we can take N epochs of size $2cN^4/\varepsilon$ each, and the probability that the process does not finish in any epoch is at most $\frac{1}{2^N}$. □

Lemma 6. *For any graph G_N with N vertices any $r \geq 1$, and a process $p \in \{Bd,\ dB\}$ there exists a vertex v such that $\rho_r^{\mathrm{p}}(G_N, v) \geq \frac{1}{N}$.*

Proof. It suffices to prove the statement for $r = 1$, since increasing the mutant fitness advantage r increases its fixation probability [6, Theorem 6].

In the neutral case ($r = 1$), we have $\sum_{v \in V} \rho_{r=1}(G_N, v) = 1$, thus there exists at least one vertex with fixation probability at least $\frac{1}{N}$. □

Note that in some cases, no starting vertex v satisfies both $\rho_r^{\mathrm{Bd}}(G_N, v) \geq 1/N$ and $\rho_r^{\mathrm{dB}}(G_N, v) \geq 1/N$ simultaneously. An example is a Star graph S_3 on $N = 3$ vertices with center c and leaves l_1, l_2 when $r = 1$. Then $\rho_r^{\mathrm{Bd}}(S_3, c) = 1/5 < 1/3$ and $\rho_r^{\mathrm{dB}}(S_3, l_1) = \rho_r^{\mathrm{dB}}(S_3, l_2) = 1/4 < 1/3$.

4.2 Construction

For given N and $\gamma \in (0, 1)$, we describe how to construct graph $A_{N,\gamma}$. We show that for some γ, this graph is an amplifier for both processes for $r \in (1, 1.2)$. The graph $A_{N,\gamma}$ has two parts. The first part is a graph $\mathcal{A}^{Bd}_{(1-\gamma)N}$ (from Lemma 2) on $(1-\gamma)N$ vertices, the second part is a graph $\mathcal{A}^{dB}_{\gamma N}$ (from Lemma 3) on γN vertices. Let ε be the smallest weight among edges when both graphs are independently scaled such that the largest edge weight is 1.

We will connect the two parts by a single edge. To that end, we select a vertex v from $\mathcal{A}^{Bd}_{(1-\gamma)N}$ such that the fixation probability starting from v in $\mathcal{A}^{Bd}_{(1-\gamma)N}$ in dB-process is at least $\frac{1}{N}$, (such vertex exists from Lemma 6). Similarly, we select a vertex v' from $\mathcal{A}^{dB}_{\gamma N}$ such that the fixation probability starting from v' in the graph under Bd-process is at least $\frac{1}{N}$, (existence follows from Lemma 6). Then, we connect v and v' by an edge of weight $w = \frac{\varepsilon^3}{N^9}$.

Finally, we scale all edges in the first part $\mathcal{A}^{Bd}_{(1-\gamma)N}$ by a factor of $\frac{\varepsilon}{N^3}$. That is, the heaviest edge in $\mathcal{A}^{dB}_{\gamma N}$ has weight 1, and the heaviest edge in $\mathcal{A}^{Bd}_{(1-\gamma)N}$ has weight $\frac{\varepsilon}{N^3}$. Observe that the scaling of edges in $\mathcal{A}^{Bd}_{(1-\gamma)N}$ does not influence the fixation time.

Before we turn to the main proof, we show several properties of the graph we $A_{N,\gamma}$ we have just constructed. The first property is that the two parts $\mathcal{A}^{Bd}_{(1-\gamma)N}$ and $\mathcal{A}^{dB}_{\gamma N}$ interact so rarely that most of the time they interact, the population on either part is already homogeneous (all mutants or all residents). Then we show Lemma 8 and Lemma 9. The lemmas show that in both processes, the probability of an individual reproducing over the edge between v and v' is unbalanced and in both processes, the individual in the respective amplifier is more likely to spread to the other graph.

Lemma 7. *For any N, γ, both processes, and a randomly placed mutant in $A_{N,\gamma}$, the probability that mutants become extinct or fixate on their part of $A_{N,\gamma}$ before any reproduction over edge v, v' is at least*

$$1 - \mathcal{O}(1/N^2).$$

Proof. First, we bound the probability that edge v, v' is selected in both processes and then we use the union bound.

For Bd, the edge v, v' is used either by (i) selecting the individual at v and spreading over v, v', or (ii) selecting the individual at v' and spreading over v', v. Event (i) happens with probability at most $\frac{r}{N+(r-1)} \cdot \frac{\varepsilon^3/N^9}{\varepsilon^3/N^9+\varepsilon^2/N^3} < \frac{r\varepsilon}{N^7}$. Event (ii) happens with probability at most $\frac{r}{N+(r-1)} \cdot \frac{\varepsilon^3/N^9}{\varepsilon^3/N^9+\varepsilon} < \frac{r\varepsilon^2}{N^{10}}$. The sum of these probabilities is at most $\frac{2r\varepsilon}{N^7}$.

For dB, the edge v, v' is used either if (i) individual at v dies and is replaced individual at v', or (ii) individual at v' dies and is replaced by individual at v. Event (i) happens with probability at most $\frac{1}{N} \cdot \frac{r\varepsilon^3/N^9}{\varepsilon^3/N^9+\varepsilon^2/N^3} < \frac{r\varepsilon}{N^7}$. Event (ii) happens with probability at most $\frac{1}{N} \cdot \frac{r\varepsilon^3/N^9}{\varepsilon^3/N^9+\varepsilon} < \frac{r\varepsilon^2}{N^{10}}$. The sum of these probabilities is at most $\frac{2r\varepsilon}{N^7}$.

From Lemma 5, we know that with high probability the process ends in $\mathcal{O}(N^5/\varepsilon)$ steps. In every step the probability of using edge v, v' is at most $\frac{2r\varepsilon}{N^7}$, that gives probability of using v, v' at most $\mathcal{O}(\frac{1}{N^2})$ at first N^5/ε steps from union bound. Since the probability that the process does not end during these steps is also in $\mathcal{O}(\frac{1}{N^2})$, we have that the randomly placed mutant resolves on one part of the graph before using edge v, v' with a probability at least $1 - \mathcal{O}(\frac{1}{N^2})$ □

Lemma 8. *In the graph $A_{N,\gamma}$ under the Bd process, if edge v, v' is used, then with probability at least $1 - \frac{r^2}{N^2}$ occupant of v spreads to v'.*

Proof. At one step, individual at v spreads to v' with probability at least $\frac{1}{rN} \cdot \frac{\varepsilon^3/N^9}{\varepsilon^3/N^9+(N-1)\cdot\varepsilon/N^3} > \frac{\varepsilon^2}{rN^8}$. Individual at v' spreads to v with probability at most $\frac{r}{N+(r-1)} \cdot \frac{\varepsilon^3/N^9}{\varepsilon^3/N^9+\varepsilon} < \frac{r\varepsilon^2}{N^{10}}$. Conditioned that the spread over v, v' happens, it is from v' to v with probability at most

$$\frac{\frac{r\varepsilon^2}{N^{10}}}{\frac{\varepsilon^2}{rN^8} + \frac{r\varepsilon^2}{N^{10}}} < \frac{r^2}{N^2}.$$

The opposite event, v spreading to v' happens with probability at least $1 - \frac{r^2}{N^2}$. □

Lemma 9. *In the graph $A_{N,\gamma}$ under the dB process, if edge v, v' is used, then with probability at least $1 - \frac{r^2}{N^2}$ occupant of v' spreads to v.*

Proof. At one step, individual at v' spreads to v with probability at least $\frac{1}{N} \cdot \frac{\varepsilon^3/N^9}{\varepsilon^3/N^9+r(N-1)\cdot\varepsilon/N^3} > \frac{\varepsilon^2}{rN^8}$. Individual at v' spreads to v with probability at most $\frac{1}{N} \cdot \frac{r\varepsilon^3/N^9}{r\varepsilon^3/N^9+\varepsilon} < \frac{r\varepsilon^2}{N^{10}}$. Conditioned that the spread over v, v' happens, it is from v to v' with probability at most

$$\frac{\frac{r\varepsilon^2}{N^{10}}}{\frac{r\varepsilon^2}{N^8} + \frac{r\varepsilon^2}{N^{10}}} < \frac{r^2}{N^2}.$$

The opposite event, v spreading to v' happens with probability at least $1 - \frac{r^2}{N^2}$. □

4.3 Proof of Amplification

Lemma 10 (Amplification under Bd)**.** *For every r and Bd updating, the fixation probability on $A_{N,\gamma}$ is at least*

$$1-\gamma-\mathcal{O}(N^{-1/3})\,.$$

Proof. For Bd, we first bound the probability that mutants conquer $\mathcal{A}^{Bd}_{(1-\gamma)N}$. For this to happen, it suffices if:

1. The initial mutant appears at the correct part of the graph (that is, $\mathcal{A}^{Bd}_{(1-\gamma)N}$).
2. In the next N^5/ε steps, the process ends in $\mathcal{A}^{Bd}_{(1-\gamma)N}$ without edge v, v' being used.
3. Mutants conquer $\mathcal{A}^{Bd}_{(1-\gamma)N}$ within N^5/ε steps.

The first condition is fulfilled with probability $1-\gamma$ since the initialization is uniformly random. The process resolves on $\mathcal{A}^{Bd}_{(1-\gamma)N}$ without edges v, v' interference with probability $1-\mathcal{O}(\frac{1}{N^2})$, from Lemma 7. If the process is finished, the mutants spread with probability at least $1-\mathcal{O}(N^{-1/3})$, from Lemma 2.

Putting these probabilities together gives a probability at least

$$(1-\gamma)\cdot\left(1-\mathcal{O}\left(N^{-2}\right)\right)\cdot\Big(1-\mathcal{O}(N^{-1/3})\Big) > 1-\gamma-\mathcal{O}(N^{-1/3})$$

that the mutants conquer $\mathcal{A}^{Bd}_{(1-\gamma)N}$.

After the graph $\mathcal{A}^{Bd}_{(1-\gamma)N}$ is occupied by mutants, we bound from below the probability that the mutants fixate in the rest of the graph. We wait until the edge (v, v') is used for reproduction, in one of its two directions. For fixation on the whole graph to occur, it suffices if:

1. The edge v, v' was used in the right direction (from v to v').
2. In the next N^5/ε steps, edge v, v' is not used for reproduction.
3. Mutants fixate on $\mathcal{A}^{dB}_{\gamma N}$ within N^5/ε steps.

The first condition happens with probability at least $1-\frac{r^2}{N^2}$, from Lemma 8. The edge v, v' is not used within N^5/ε steps with probability at least $1-\mathcal{O}(N^{-2})$, again from Lemma 7. If both of those occur, the mutants fixate with probability at least $\frac{1}{N}$, from Lemma 6 and since the process finishes within N^5/ε steps with probability at least $1-2^{-N}$, by Union Bound the fixation probability is at least $\frac{1}{N}-2^{-N}$.

This gives the probability at least

$$\left(1-\frac{r^2}{N^2}\right)\cdot\left(1-\mathcal{O}(N^{-2})\right)\cdot\left(\frac{1}{N}-2^{-N}\right) > \frac{1}{N}-\frac{1}{N^2}$$

that if the edge (v, v') is used, the process finishes with mutant fixation on the whole graph without edge (v, v') being used again.

In contrast, if condition 1. fails, that is, the edge (v, v') is instead used in the wrong direction (from v' to v), we declare a failure (even though some of those evolutionary trajectories might eventually lead to mutant fixation). By Lemma 8, this happens with probability at most $\frac{r^2}{N^2}$. Similarly, we declare a failure if condition 2. fails, that is, when the edge (v, v') is used (in either direction) during the N^5/ε steps, potentially interrupting the process. Note that when condition 3. fails, that is, the mutants do not fixate in $\mathcal{A}^{dB}_{\gamma N}$ (but the edge v, v' is not used), we are in the same state as before, where we can compute the fixation versus failure probability.

The failure probability is in $\mathcal{O}(N^{-2})$, the immediate fixation probability is at least $\frac{1}{N}-\frac{1}{N^2}$, otherwise, we can retry. This gives the fixation probability at least

$$\frac{\frac{1}{N}-\frac{1}{N^2}}{\mathcal{O}(N^{-2})+\left(\frac{1}{N}-\frac{1}{N^2}\right)} = 1-\mathcal{O}\left(\frac{1}{N}\right).$$

Overall, the fixation probability of a randomly placed mutant on $A_{N,\gamma}$ is thus at least

$$\Big(1-\gamma-\mathcal{O}(N^{-1/3})\Big)\cdot\left(1-\mathcal{O}\left(\frac{1}{N}\right)\right) \geq 1-\gamma-\mathcal{O}(N^{-1/3}).$$

□

Lemma 11 (Amplification under dB)**.** *For every r and dB updating, the fixation probability on $A_{N,\gamma}$ is at least*

$$\left(\frac{1}{2}\gamma(1-r^{-3}) - \mathcal{O}(N^{-1})\right) .$$

Proof. For dB, we proceed similarly as in the previous lemma. First, we again bound the probability that mutants conquer $\mathcal{A}^{dB}_{\gamma N}$. For this to happen, it suffices if:

1. The initial mutant appears at the correct part of the graph: $\mathcal{A}^{dB}_{\gamma N}$.
2. In the next N^5/ε steps, the process ends in $\mathcal{A}^{dB}_{\gamma N}$ without edge v, v' being used.
3. Mutants conquer $\mathcal{A}^{dB}_{\gamma N}$ within N^5/ε steps.

The first condition is fulfilled with probability γ since the initialization is uniformly random. The process resolves on $\mathcal{A}^{dB}_{\gamma N}$ without edges v, v' interference with probability $1 - \mathcal{O}(\frac{1}{N^2})$, from Lemma 7. If the process has resolved on $\mathcal{A}^{dB}_{\gamma N}$, the mutants conquer it with probability at least $\frac{N}{2N+1} \cdot \frac{1-\frac{1}{r^3}}{1-\frac{1}{r^{3N}}} - \mathcal{O}(2^{-N})$, from Lemma 3 and Union Bound.

Putting these probabilities together gives a probability at least

$$\gamma \cdot \left(1 - \mathcal{O}\left(N^{-2}\right)\right) \cdot \left(\frac{N}{2N+1} \cdot \frac{1-\frac{1}{r^3}}{1-\frac{1}{r^{3N}}} - \mathcal{O}(2^{-N})\right) > \frac{1}{2}\gamma(1-r^{-3}) - \mathcal{O}(N^{-1})$$

that the mutants conquer $\mathcal{A}^{dB}_{\gamma N}$.

After the graph $\mathcal{A}^{dB}_{\gamma N}$ is occupied by mutants, we bound the probability that the mutants fixate in the rest of the graph. Conditioned on the fact that the edge v, v' is used, it happens when

1. The edge v, v' was used in the right direction (from v' to v).
2. In the next N^5/ε steps, edge v, v' is not used.
3. Mutants fixate on $\mathcal{A}^{Bd}_{(1-\gamma)N}$ within N^5/ε steps.

If the edge v, v' is used in the wrong direction, we call it a fail, this happens with probability at most $\frac{r^2}{N^2}$, from Lemma 9 (if the edge is used).

The first condition happens with probability at least $1 - \frac{r^2}{N^2}$, from Lemma 9. The edge v, v' is not used within N^5/ε steps with probability at least $1 - \mathcal{O}(N^{-2})$, again from Lemma 7. The mutants fixate with probability at least $\frac{1}{N}$, from Lemma 6 and since process finishes within N^5/ε steps with probability at least $1 - 2^{-N}$, the fixation is at least $\frac{1}{N} - 2^{-N}$.

This gives the probability at least

$$\left(1 - \frac{r^2}{N^2}\right) \cdot \left(1 - \mathcal{O}(N^{-2})\right) \cdot \left(\frac{1}{N} - 2^{-N}\right) > \frac{1}{N} - \frac{1}{N^2}$$

that if the edge v, v' is used, the process finishes without edge v, v' being used again.

However, when the mutants do not fixate in $\mathcal{A}^{Bd}_{(1-\gamma)N}$ (but the edge v, v' is not used), we are in the same state as before, where we can compute the fixation versus fail probability.

The fail probability is in $\mathcal{O}(N^{-2})$, the immediate fixation probability is at least $\frac{1}{N} - \frac{1}{N^2}$, otherwise, we can retry. This gives total fixation $1 - \mathcal{O}(\frac{1}{N})$.

Overall, the fixation probability of a randomly placed mutant on $A_{N,r}$ is at least

$$\left(\frac{1}{2}\gamma(1-r^{-3}) - \mathcal{O}(N^{-1})\right) \cdot \left(1 - \mathcal{O}(\frac{1}{N})\right) \geq \left(\frac{1}{2}\gamma(1-r^{-3}) - \mathcal{O}(N^{-1})\right) .$$

□

The following theorem shows that for a particular γ, our construction is an amplifier for both processes for $r \in (1, 1.2)$.

Theorem 3 (Simultaneous Bd- and dB-amplifier). *For every large enough population size N, for graph $A_{N,\gamma}$, where $\gamma = \frac{2 \cdot 1.2^3}{3 \cdot 1.2^3 - 1} = 0.826004$ we have*

$$\rho_r^{\mathrm{Bd}}(A_{N,\gamma}) > \rho_r^{\mathrm{Bd}}(K_N) \quad \textit{and} \quad \rho_r^{\mathrm{dB}}(A_{N,\gamma}) > \rho_r^{\mathrm{dB}}(K_N)$$

for every $r \in (1, 1.2)$.

Proof. We know that $\rho_r^{\mathrm{Bd}}(K_N) = \frac{1-r^{-1}}{1-r^{-N}}$ and $\rho_r^{\mathrm{dB}}(K_N) = \frac{N-1}{N} \frac{1-r^{-1}}{1-r^{-N+1}}$. Setting N so big that $\frac{1}{1-r^{-N+1}} < 1.00001$, we have that $\rho_r^{\mathrm{Bd}}(K_N) < (1-r^{-1}) \cdot 1.00001 < 0.16667$ and $\rho_r^{\mathrm{dB}}(K_N) < (1-r^{-1}) \cdot 1.00001$.

From Lemma 10, plugging γ, we have that the fixation probability is at least $1 - 0.826004 - \mathcal{O}(N^{-1/3}) = 0.173996 - \mathcal{O}(N^{-1/3})$ for the Birth-death process which is bigger than the maximal fixation probability for K_N (0.16667).

From Lemma 11, plugging γ, we have that the fixation probability is at least $\left(\frac{1}{2} 0.826004(1-r^{-3}) - \mathcal{O}(N^{-1})\right)$ for the death-Birth process. We have

$$\begin{aligned}
(1-r^{-1}) \cdot 1.00001 &< \left(\frac{1}{2} 0.826004(1-r^{-3}) - \mathcal{O}(N^{-1})\right) \\
1.00001 &< \left(\frac{1}{2} 0.826004(1+r^{-1}+r^{-2})\right) - \mathcal{O}(N^{-1}) \\
1.00001 &< \left(\frac{1}{2} 0.826004 \cdot 2.52778\right) - \mathcal{O}(N^{-1}) \\
1.00001 &< 1.04 - \mathcal{O}(N^{-1}),
\end{aligned}$$

which proves the theorem. □

The following theorem shows how to choose γ to achieve the best amplification so that the fixation probability of the amplifier is at least 1.04 times better than the complete graph for both processes.

Theorem 4 (Optimal Bd- and dB-amplifier). *For any $r \in (1, 1.2)$, and every large enough population size N, for graph $A_{N,\gamma}$, where $\gamma = \frac{2r^3}{3r^3-1}$ we have*

$$\rho_r^{\mathrm{Bd}}(A_{N,\gamma}) > X \cdot \rho_r^{\mathrm{Bd}}(K_N) \quad \textit{and} \quad \rho_r^{\mathrm{dB}}(A_{N,\gamma}) > X \cdot \rho_r^{\mathrm{dB}}(K_N).$$

for $X = 1.04$.

Proof. Again, $\rho_r^{\mathrm{Bd}}(K_N) = \frac{1-r^{-1}}{1-r^{-N}}$ and $\rho_r^{\mathrm{dB}}(K_N) = \frac{N-1}{N} \frac{1-r^{-1}}{1-r^{-N+1}}$. Setting N so big that $\frac{1}{1-r^{-N+1}} < 1.00001$, we have that $\rho_r^{\mathrm{Bd}}(K_N) < (1-r^{-1}) \cdot 1.00001$ and $\rho_r^{\mathrm{dB}}(K_N) < (1-r^{-1}) \cdot 1.00001$.

From Lemma 10, plugging γ, we have that the fixation probability is at least $\frac{r^3-1}{3r^3-1} - \mathcal{O}(N^{-1/3})$ for the Birth-death process. We have

$$\begin{aligned}
(1-r^{-1}) \cdot 1.00001 \cdot X &< \frac{r^3-1}{3r^3-1} - \mathcal{O}(N^{-1/3}) \\
1.00001 \cdot X &< \frac{r(1+r+r^2)}{3r^3-1} - \mathcal{O}(N^{-1/3}) \\
1.00001 \cdot X &< 1.04398 - \mathcal{O}(N^{-1/3}).
\end{aligned}$$

From Lemma 11, plugging γ, we have that the fixation probability is at least $\left(\frac{1}{2} \frac{2r^3}{3r^3-1}(1-r^{-3}) - \mathcal{O}(N^{-1})\right)$ for

the death-Birth process. We have

$$\begin{aligned}
(1-r^{-1})\cdot 1.00001\cdot X &< \left(\frac{1}{2}\frac{2r^3}{3r^3-1}(1-r^{-3})-\mathcal{O}(N^{-1})\right)\\
1.00001\cdot X &< \left(\frac{1}{2}\frac{2r^3}{3r^3-1}(1+r^{-1}+r^{-2})-\mathcal{O}(N^{-1})\right)\\
1.00001\cdot X &< \left(\frac{r(1+r+r^2)}{3r^3-1}-\mathcal{O}(N^{-1})\right)\\
1.00001\cdot X &< 1.04398-\mathcal{O}(N^{-1})\,.
\end{aligned}$$

□

References

[1] Benjamin Allen and Alex McAvoy. A mathematical formalism for natural selection with arbitrary spatial and genetic structure. *Journal of mathematical biology*, 78(4):1147–1210, 2019.

[2] Benjamin Allen, Christine Sample, Robert Jencks, James Withers, Patricia Steinhagen, Lori Brizuela, Joshua Kolodny, Darren Parke, Gabor Lippner, and Yulia A Dementieva. Transient amplifiers of selection and reducers of fixation for death-birth updating on graphs. *PLoS computational biology*, 16(1):e1007529, 2020.

[3] Tibor Antal, Sidney Redner, and Vishal Sood. Evolutionary dynamics on degree-heterogeneous graphs. *Physical review letters*, 96(18):188104, 2006.

[4] M Broom, C Hadjichrysanthou, J Rychtář, and BT Stadler. Two results on evolutionary processes on general non-directed graphs. *Proceedings of the Royal Society A: Mathematical, Physical and Engineering Sciences*, 466(2121):2795–2798, 2010.

[5] Josep Díaz, Leslie Ann Goldberg, George B Mertzios, David Richerby, Maria Serna, and Paul G Spirakis. Approximating fixation probabilities in the generalized moran process. *Algorithmica*, 69:78–91, 2014.

[6] Josep Díaz, Leslie Ann Goldberg, David Richerby, and Maria Serna. Absorption time of the moran process. *Random Structures & Algorithms*, 49(1):137–159, 2016.

[7] Loke Durocher, Panagiotis Karras, Andreas Pavlogiannis, and Josef Tkadlec. Invasion dynamics in the biased voter process. In *Proceedings of the Thirty-First International Joint Conference on Artificial Intelligence*, pages 265–271, 2022.

[8] Leslie Ann Goldberg, John Lapinskas, Johannes Lengler, Florian Meier, Konstantinos Panagiotou, and Pascal Pfister. Asymptotically optimal amplifiers for the moran process. *Theoretical Computer Science*, 758:73–93, 2019.

[9] Zhengyan Lin. *Probability inequalities.* Springer, 2010.

[10] Wes Maciejewski. Reproductive value in graph-structured populations. *Journal of Theoretical Biology*, 340:285–293, 2014.
